# What Race Am I? Factors Associated with Racial Self-classification Among U.S. Latinx Adults

**DOI:** 10.1017/S1742058X25000050

**Published:** 2025

**Authors:** Victor Figuereo, Robert Rosales, David T. Takeuchi, Rocío Calvo

**Affiliations:** 1 School of Social Work, University of Pittsburgh, Pittsburgh, PA, USA; 2 School of Public Health, Brown University, Providence, RI, USA; 3 School of Social Work, University of Washington, Seattle, WA, USA; 4 School of Social Work, Boston College, Chestnut Hill, MA, USA

**Keywords:** Race, Ethnicity, Latinx, AfroLatinx, Socioeconomic Status, Immigration, Acculturation, Racialization

## Abstract

This study examined how immigrant status and socioeconomic status influence racial self-classification among U.S. Latinx adults aged eighteen and older across multiple nationalities. Using data from the 2010–2018 National Health Interview Survey, we analyzed a nationally representative sample of Mexican, Cuban, Puerto Rican, Dominican, and Central/South American adults (N = 41,133) who identified as White, Black, or Another race. Socioeconomic status was measured using a composite index of income-to-poverty ratio, education, employment status, and homeownership. Multinomial logistic regressions and average marginal effects revealed significant heterogeneity in examined predictors of racial identity. U.S.-born Latinx adults, particularly Puerto Ricans and Central/South Americans, had higher probability of identifying as Black compared to recent immigrants. Latinx adults with low and middle socioeconomic status backgrounds were more likely to identify as Black or Another race across most nationality groups. Findings highlight the complexity of Latinx racial identity, whereby Latinxs may experience racialization differently depending on indicators of acculturation and socioeconomic status. The inclusion of multidimensional measures of race, such as skin color and street race, in future research is needed to better understand Latinx racial identity formation. Findings inform interventions to address race-related stress and anti-Blackness, particularly among AfroLatinx populations, and provide considerations for improving race data collection practices, such as those impacted by recent federal policy changes to the U.S. Census.

## Introduction

Race is generally accepted as socially constructed and malleable, frequently defined by political decisions and public audiences. This sociological fact is never more apparent than with the racial self-classifications of Hispanic and Latinx individuals (from here on these will be referred to as Latinx). Racial self-classification refers to the racial options one self-classifies into when prompted, such as on a survey like the Census (Roth 2016). The U.S. federal government defines “Hispanic and Latino” as an ethnic group that can be of any race. According to the last decennial Census, 42% of Latinx adults self-classified as “Some Other Race” (from here on will be referred to as Another race^1^), 20% self-classified as “White”, and slightly less than 2% of Latinx adults indicated their race was “Black” (Census 2021). The population of Latinx people who identify as Another race or Black have significantly increased since previous census counts. AfroLatinx identity^2^ has increased by over 121% between 2000 and 2019 and Another race is now the largest racial representation of Latinxs in the United States (Galdámez et al., 2023).

These trends raise important questions about the factors that influence racial self-classification among Latinx adults. Given how heterogenous Latinxs are in their nationality (i.e., country of origin), immigrant status (i.e., nativity and length of stay in the U.S.), and socioeconomic circumstances (i.e., income, education), how do these factors influence racial identity and the race options one opts into? While acculturation, socioeconomic status (SES), and nationality have been identified as predictors of White and Another racial self-classification (e.g., Filindra and Kolbe, 2022; Golash-Boza and Darity, 2008; Rodriguez et al., 2013), findings are mixed, and little is known about how these factors predict Black racial self-classification.

The present study seeks to fill this gap by examining how immigrant status and SES predict Black or Another race self-classification (vs. White) across multiple nationality groups among Mexican, Puerto Rican, Cuban, Dominican, Central, and South American adults. Using recent national data, this study provides an updated understanding of these predictors and contributes to ongoing discussions about the sociopolitical and cultural dynamics shaping Latinx racial identity.

## Theoretical Considerations: Predictors of Latinx Racial Self-Classification

Acculturation refers to the process through which Latinx individuals adapt to the cultural norms, values, and social practices of the dominant American culture while retaining aspects of their original cultural identity (Berry 2005; Schwartz et al., 2010). Acculturation has been measured in various ways, typically through constructs such as nativity status and length of U.S. residence. Assimilation is a broader process that can result in the loss of original cultural identity as individuals and groups adopt the dominant culture’s norms, values, and practices to a degree where they become indistinguishable from the majority population (Gordon 1964; Waters and Jiménez, 2005). Under traditional assimilation theory, researchers predicted Latinx individuals who have lived in the United States for longer periods and who earn higher income and education will experience social Whitening, be accepted into the White mainstream, and become White-identifying individuals (e.g., Yancey 2003). A literature review of factors that influence racial self-classifications partially support and contradict this theorized effect (Rodriguez et al., 2013).

### The Role of Socioeconomic Status on Racial Self-Classification

According to analyses from the 2002 National Latino Survey (NLS), Latinx individuals who attended college or graduated and with a family income of over $50,000 were more likely to self-classify as White (Golash-Boza and Darity, 2008). Afro-Latinxs experience lower SES conditions (income, poverty), report greater discrimination, and experience worse health outcomes (hypertension, and low-birth weight) compared to their WhiteLatinx counterparts (Borrell 2009; Borrell and Crawford, 2006; Borrell and Dallo, 2008; Cuevas et al., 2016; Mena et al., 2019; LaVeist-Ramos et al., 2012; Logan 2003). Therefore, Latinxs with higher income may be more attracted to Whiteness and White identity to distance themselves from stigmatized identities that experience inequity, such as Black and Another race. More recent research aligns with these findings. Alexandra Filindra and Melanie Kolbe (2022) found that higher income continues to be associated with identifying as White, while education is increasingly linked to identifying as Latinx.

Though there are no studies that have examined predictors of Black racial identity among Latinx adults, Elizabeth Hordge-Freeman and Edlin Veras’ qualitative study (2020) on the identity formation of Afro-Latinx individuals provides insight into the role of higher education. The authors conducted a survey with ninety-four self-identified Afro-Latinxs and in-depth interviews with selected respondents. The findings reveal that Afro-Latinx identity formation is seldom supported by racial affirmation from families. Instead, Afro-Latinxs frequently encounter colorism and negative appraisals of Black racialized features within their families. This lack of familial support, coupled with the stigmatization of Blackness, leads to early racial socialization marked by ethnoracial dissonance. However, the researchers found that exposure to college environments has provided some Afro-Latinxs with alternative and positive constructions of Blackness, facilitating the formation of Black and Afro-Latinx identity. Though earlier evidence suggests higher income is associated with White identity and education is associated with Latinx identity, Hordge-Freeman and Veras’ study also indicates higher education could be associated with Black/Afro-Latinx identity.

### The Role of Location on Racial Self-Classification

Clara E. Rodriguez and colleagues (2013) indicate that location matters in racial self-classification. Latinx adults differ in where they live in the United States. While some reside in ethnic enclaves, others live in new destinations, which disperse Latinx adults across all the major U.S. regions. One study found that living in the Southwestern region of the United States increased the likelihood of classifying as “Another race” and decreased the likelihood of classifying into traditional U.S. racial categories when compared to living in the U.S. South (Frank et al., 2010). Another study found that Mexicans living in Los Angeles, CA were more likely to classify as “Another race” than Mexicans living in San Antonio, TX (Ortiz and Telles, 2012). These findings may be explained by the social, cultural, and political atmosphere of these locations.

Ivelisse Cuevas-Molina (2023) suggests that White identification among Latinx individuals may be influenced by the racial attitudes and partisanship prevalent in their local social environment. Specifically, the study highlights that higher identification with Whiteness and negative racial attitudes are associated with stronger Republican partisanship. This implies that Latinx individuals living in predominantly Republican areas may be more likely to identify as White because it aligns with socially acceptable political norms in these contexts. Building on this, Jorge Ballinas and James Bachmeier (2020) found that Mexican-origin individuals in Texas are significantly more likely to identify as White compared to their counterparts in California. This disparity is attributed to Texas’s historical and social context, where Whiteness carries a distinctive social value, influencing racial self-identification among Mexican-origin populations. Additionally, Rudy Alamillo (2020) examined Hispanic support for Donald Trump and discovered that denial of racism is a strong predictor of such support, surpassing traditional factors like party identification and ideology. This suggests that among some Latinx individuals, a denial of systemic racism correlates with both a stronger alignment with Republican partisanship and an increased likelihood of identifying as White. The longer individuals remain in a social environment and adapt to its cultural norms, the more this process may be reinforced. Rodriguez and colleagues (2013) state that acculturation is a predictor of Latinx racial self-classification.

### The Role of Acculturation on Racial Self-Classification

Researchers found that less acculturated Latinx adults (first-generation status, Spanish speaking) were more likely to choose “Another race” (Lee and Tafoya, 200; Rodriguez 2000; Tafoya 2004; Vaquera and Kao, 2006).

Contradicting findings reveal the opposite direction, whereby more acculturated Latinx adults may also be more likely to select “Another race”. These studies show that greater length of residence in the United States is related to Latinx people classifying as “Another” over White (Frank et al., 2010; Stokes-Brown 2012; Vargas-Ramos 2015). Qualitative data using interviews with Puerto Ricans, Dominicans, and Ecuadorians also support this direction indicating that those born in the United States and those with high incomes reported “Another” as their race (Rodriguez 2000).

Scholars suggest that Latinx adults who have recently immigrated and not yet exposed to the White-Black binary system of race may still use their country of origin’s racial schemas (Roth 2012). They may use the “Another race” option to assert their understanding of race based on ethnicity (i.e., “Latinx”), national identity (e.g., “I am Dominican”) or a range of skin color and other phenotype characteristics (e.g., “Negra/o”, “Morena/o”, “India/o”, “Mestiza/o”, “Blanca/o”). However, the positive association between acculturation and Another race may be because longer exposure to the United States is associated with greater perceived discrimination (Ortiz and Telles, 2012; Salas-Wright et al., 2020). Latinx adults may select “Another” as they cope through discrimination and realize they are not accepted and treated as White. In response to experiences of discrimination, a qualitative study found that some Mexican American adults in Texas asserted their cultural heritage by racially classifying as “Another” and claiming a “Hispanic” identity (Dowling 2014).

### The Role of Nationality on Racial Self-Classification

Finally, Rodriguez and colleagues (2013) assert that variation in nationality among Latinx individuals must also be acknowledged and taken into consideration when examining racial reporting patterns. Nationality refers to the country or region where a person was born or from which their ancestors originate. This concept is important in understanding the cultural and historical contexts that shape individuals’ identities and experiences (Gordon 1964). U.S. Latinx adults have ancestry from over twenty Latin American and Caribbean lands and countries. The relationship between immigration, SES factors, and self-classification as Black and Another race may vary among Latinx nationality due to members of one nationality group being racialized differently than others. According to the segmented racialization hypothesis (Henry-Sanchez and Geronimus, 2013), Mexicans may often be racialized as “non-White other”, Cubans as White, and Puerto Ricans as Black. This racialization may be influenced by each group’s different sociodemographic profiles, such as skin color; SES; immigration, geographic, and political patterns; and histories. Different rates of homeownership and behavioral health outcomes may reflect this racialization process. Puerto Ricans and Dominicans are less likely and Cubans are more likely to own a home than Mexicans (Martinez and Aja, 2021).

Research has yet to assess how acculturation may differ in the context of national origin using a nationally representative sample. Understanding how segmented racialization processes vary by nationality may help elucidate the relationships between immigrant status, SES, and racial self-classification.

### The Role of Skin Color on Racial Self-Classification

It is important to note that one form of discrimination that social Whitening and traditional assimilation hypotheses of Latinx racial identity have not accounted for is skin color discrimination.

Colonization in Latin America and the Caribbean developed pigmentocracies and skin color hierarchies that stigmatized darker skinned individuals and placed them at the bottom (Telles 2014). Scholars have highlighted the impact of colorism on racial self-identity (Frank et al., 2010; Golash-Boza and Darity, 2008; Stokes-Brown 2012). Latinx adults who experience or perceive discrimination based on their skin color are more likely to self-classify as Black than White (Stokes-Brown 2012). Another impact of colonization on Latinx racial identity today is the internalization of colorism and racial attitudes/ideologies that favor Whiteness, such as *Mestizaje Racial Ideologies.* ^3^ For example, some individuals from Latin American countries with many afro-descendants, such as Dominican Republic, prefer to racially identify with their nationality and purport against Black identity despite having Black racialized phenotype features (e.g., darker skin) because of the stigma associated with Blackness (Adames et al., 2021; Torres-Saillant 2010).

## Present Study

While previous research has explored acculturation and SES as predictors of racial identity among Latinx individuals, these studies have often lacked a focus on Black racial identity and have not fully accounted for variations across multiple Latinx nationality groups. The present study aims to address these gaps by examining whether Latinx racial self-classification (i.e., Black or Another race) is influenced by immigrant status and socioeconomic status (SES). This study focused on Mexican, Puerto Rican, Cuban, Dominican, Central and South American adults while controlling for age, sex, marital status, and U.S. region.

This study will address the following research questions:

Research Question 1: Is immigrant status associated with Black or Another race racial self-classification?

Research Question 2: Is SES associated with Black or Another race racial self-classification?

Racial and ethnic identity development research on non-Latinx African Americans and Latinxs has had a significant impact on understanding the effects of racism and race-related stressors on their health and mental health (e.g., Sellers et al., 1998; Brittian et al., 2015; Umaña-Taylor et al., 2018). Given the impact Latinx racial identity has on health and well-being, identifying what predictors of racial self-classification may differ between nationality groups may also provide insight into the effects of racism on the psychological wellbeing of Latinxs and help better understand the mechanisms of this pathway. This is important more than ever because Afro-Latinx identity has increased by over 100% in the last two decades and Another race is now the largest racial representation of Latinxs in the United States (Galdámez et al., 2023). By focusing on diverse Latinx nationalities and using recent national data, this study aims to contribute to a better understanding of the predictors of racial self-classification and the implications they may have on the broader social, psychological, and health-related experiences of Latinx individuals.

Skin color was not available in the dataset due to its absence in the dataset analyzed in this study. Latinxs with darker skin report more discrimination and Latinxs who have experienced more discrimination are more likely to believe skin color shapes their lives, including racial identity (Noe-Bustamante et al., 2021). Despite the essential role skin color plays in the racial identification of Latinxs, several challenges when measuring skin color may contribute to the under-collection of skin color data in national surveys like the one used in this study, including grappling with how to accurately and reliability measure it (e.g., self-report vs. interviewer ascribed vs. spectrometers, with or without palettes or guides, which body part) and the cost of using spectrometers and color palettes (Dixon and Telles, 2017). We acknowledge that skin color may affect the results of this study, thus we discuss our findings considering this limitation.

## Method

### Data and Sample

Extant data originates from the National Health Interview Survey (National Center for Health Statistics 2019). The National Health Interview Survey (NHIS) uses a multistage sampling approach to survey randomly selected U.S. households, families, adults, and children on their health status annually. The NHIS oversamples racially and ethnically groups and maintains an 80% response rate. To increase the number of underrepresented Latinx nationality and racial groups (i.e., Dominicans and AfroLatinxs), we combined the last nine waves of data, from 2010–2018, in which specific nationality group data was publicly available. Each annual dataset we merged is publicly available on the NCHS NHIS data archive webpage (NCHS 2019).^4^ We employed merging techniques recommended by NHIS using their survey description document (NCHS 2019).^5^ Our final sample (N= 41,133) consisted of Mexican (n= 25,912), Cuban (n= 2261), Puerto Rican (n= 4453), Dominican (n= 1500), Central/South American (n= 7007) adults (eighteen years and older) who racially self-classified as White (n= 24,933), Black (n= 902), or Another race (n= 15,298).

### Dependent Variable: Racial self-classification

Racial self-classification is the race an individual selects from a closed-ended question on a survey with a set number of options, like the Census (Roth 2016). The NHIS uses this dimension of race and instructs respondents to select among the following categories: 1) White, 2) Black or African American, 3) American Indian & Alaskan Native, 4) Asian, 5) Another race. To represent the three most frequent races respondents selected, we recoded the race variable (0 = White, 1 = Black, 2 = Another race). Additional racial groups (i.e., Asian, Alaskan and Indian American Native) were omitted from this study due to low sample size among some nationality groups (n<10).

### Independent Variable: Immigrant Status

Similar to previous studies (e.g., Murillo et al., 2019), we merged the variables, nativity and length of U.S. residence, to create the variable: immigration status (0= recent immigrant or less than ten years of U.S. residence, 1= long-term immigrant or more than ten years of U.S. residence, 2= U.S.-born). For Puerto Rican respondents, immigrant status refers to whether respondents who were born in Puerto Rico and recently migrated to continental United States, including any of the fifty U.S. states or Washington DC (0), respondents who were island-born and have been living in the continental United States for more than ten years (1), or was born in the continental U.S. (2).

### Independent Variable: Socioeconomic Status

We created an SES index according to the methodology outlined by the Bureau of Justice and Statistics (Berzofsky et al., 2014, Buder et al., 2023). This index combines three SES indicators: family income to poverty ratio (0 =<100% of Federal Poverty Level (FPL), 1= 100% -199% of FPL, 2= 200%-399% of FPL, 3= 400% or more of FPL), education (0= less than high school, 1= high school, some college 2= bachelor’s degree, 3= master’s, professional, doctorate degree), employment status (0= unemployed, 1= employed), and house tenure (0=rent, 1=own). The scores for these indicators were summed to create a composite SES score ranging from 0 to 8. We then recoded the composite score into three SES classes: 0-2 (low SES), 3-5 (middle SES), and 6-8 (high SES). By combining these indicators, we aimed to create a comprehensive measure of SES that captures multiple dimensions of socioeconomic position.

### Subgroup Variable: Nationality

The variable we did subgroup analysis with was nationality, (0= Mexican, 1= Cuban, 2= Puerto Rican, 3= Dominican, 4= Central/South American).

### Control Variables

We controlled for ethnic group, (0= Mexican, 1= Cuban, 2= Puerto Rican, 3= Dominican), and U.S. region (0= West, 1= Midwest, 2= South, 3= Northeast) to address potential bias from variables linked to racial self-classification in previous studies (Frank et al., 2010; Henry-Sanchez and Geronimus, 2013). Additionally, we controlled for age (0= eighteen to twenty-five, 1= twenty-six to thirty-four, 2= thirty-five to forty-nine, 3= fifty to sixty-four, 4= sixty-five and older, sex^6^ (0= male, 1= female), marital status (0= single, 1= cohabitating or married), employment status (0= employed, 1= unemployed), and NHIS survey year.

### Analytic Plan

To describe characteristics of the sample, we computed unweighted totals and weighted percentages among the pan-ethnic sample (i.e., Latinx adults from all national origins) and by racial self-classification using Stata Statistical Software 17 SE (StataCorp, 2021), (see Table 1). By computing chi-square tests, we estimated the bivariate associations between independent, subgroup, and control variables and the dependent variable (see Table 1). We chose chi-square tests due to their suitability for categorical data.

**Table 1.** Characteristics by Racial Self-Classification in Frequency (n) and Percentages (%) with Bivariate Associations

Variable	Total (N=41,133)	White (n=24,933)	Black (n=902)	Another Race (n=15,298)	p-value
**Dependent Variable**					
**Racial Self-Classification**					–
White	24933 (60.5)	–	–	–	
Black	902 (2)	–	–	–	
Another race	15298 (37.5)	–	–	–	
**Independent Variables**					
**Immigrant Status**					***
Recent Immigrant	4198 (10.1)	2452 (9.9)	90 (9.6)	1656 (10.4)	
Long-term Immigrant	20410 (48.6)	11505 (44.9)	348 (39.8)	8557 (55.0)	
US-born	16146 (41.4)	10760 (45.3)	461 (50.6)	4925 (34.6)	
**SES Index Class**					***
Low SES	15638 (33.4)	8707 (30.1)	352(32.8)	6579 (38.7)	
Middle SES	18693 (51.3)	11590 (52.2)	376 (47.3)	6727 (50.1)	
High SES	5167 (15.3)	3643 (17.7)	133 (19.9)	1391 (11.2)	
**Subgroup Variable**					
**Nationality**					***
Mexican	25912 (63.8)	15671 (63.0)	147 (16.4)	10094 (67.5)	
Cuban	2261 (4.7)	2008 (6.9)	84 (7.3)	169 (1.0)	
Puerto Rican	4453 (10.2)	2702 (10.9)	326 (35.8)	1425 (7.9)	
Dominican	1500 (3.6)	538 (2.2)	168 (19.9)	794 (4.9)	
Central/South American	7007 (17.7)	4014 (17.0)	177 (20.6)	2816 (18.7)	
**Control Variables**					
**Age**					***
18–25	6186 (19.4)	3672 (19.0)	159 (20.5)	2355 (19.9)	
26–34	8762 (21.5)	5174 (21.3)	217 (23.7)	3371 (21.8)	
35–49	13104 (31.0)	7652 (30.1)	269 (28.6)	5183 (32.6)	
50–64	7732 (18.6)	4779 (19.0)	152 (18.1)	2801 (18.0)	
65 and older	5349 (9.5)	3656 (10.7)	105 (9.0)	1588 (7.7)	
**Sex^6^**					0.41
Male	18191 (50.1)	10911 (49.9)	379 (47.6)	6901 (50.5)	
Female	22942 (49.9)	14022 (50.1)	523 (52.4)	8397 (49.5)	
**Education**					***
Less than High School	14725 (33.2)	8118 (29.8)	211 (20.8)	6396 (39.3)	
High School/Some College	20569 (52.9)	12805 (54.2)	515 (59.2)	7249 (50.5)	
Bachelor’s Degree	3886 (9.9)	2672 (11.3)	121 (13.8)	1093 (7.4)	
Master’s/Professional/Doctorate	1616 (4.0)	1165 (4.7)	51 (6.3)	400 (2.8)	
**Poverty Status**					***
100% or less	11527 (22.6)	6583 (21.1)	272 (23.3)	4672 (25.0)	
101%–200% of FPL	12031 (29.2)	7045 (27.7)	243 (26.0)	4743 (31.7)	
201%–400% of FPL	10834 (29.2)	6772 (29.7)	222 (27.8)	3840 (28.5)	
401% or Greater	6509 (19.0)	4397 (21.5)	160 (23.0)	1952 (14.7)	
**Employment**					0.911
Unemployed	15848 (35.6)	9698 (35.7)	355 (36.1)	5795 (35.5)	
Employed	25260 (64.4)	15218 (64.3)	547 (63.9)	9495 (64.5)	
**Home Tenure**					***
Rented	22182 (49.3)	12227 (44.6)	629 (66.2)	9326 (55.9)	
Owned	17881 (50.7)	12022 (55.4)	241 (33.8)	5618 (44.1)	
**Marital Status**					***
Single	19009 (39.5)	11336 (38.2)	594 (55.1)	7079 (40.7)	
Cohabitating/Married	22055 (60.5)	13556 (61.8)	306 (44.9)	8193 (59.3)	
**US Region**					***
West	16693 (39.9)	8660 (33.4)	129 (13.3)	7904 (51.8)	
South	15183 (37.1)	11251 (45.5)	341 (37.6)	3591 (23.5)	
Midwest	3636 (9.1)	2166 (8.8)	101 (12.5)	1369 (9.4)	
Northeast	5621 (13.8)	2856 (12.2)	331 (36.5)	2434 (15.2)	
**NHIS Year**					0.068
2010	4796 (9.8)	2695 (9.1)	82 (7.7)	2019 (11.0)	
2011	5396 (9.9)	3115 (9.6)	100 (9.0)	2181 (10.5)	
2012	5422 (10.7)	3332 (11.0)	106 (8.5)	1984 (10.4)	
2013	5523 (11.0)	3346 (11.1)	112 (10.6)	2065 (11.0)	
2014	5527 (11.0)	3295 (10.8)	124 (10.9)	2108 (11.4)	
2015	5101 (11.4)	3083 (11.6)	122 (12.5)	1896 (11.2)	
2016	3479 (11.8)	2214 (11.9)	98 (13.7)	1167 (11.6)	
2017	2961 (11.9)	1979 (12.3)	81 (13.4)	901 (11.1)	
2018	2928 (12.5)	1874 (12.8)	77 (13.6)	977 (11.9)	

Data Source: NCHS, National Health Interview Surveys, 2010–2018.

Note: W= Weighted Percentages; * p<0.05; ** p<0.01; *** p<0.001.

Afterwards, we used multinomial logistic regressions to estimate the associations between the independent variables: immigrant status and SES, the subgroup variable: nationality, and the dependent variable: racial self-classification, while controlling for age, sex, U.S. region, marital status, and NHIS survey year. In this study, the dependent variable has three outcomes, White, Black, and Another Race. Multinomial logistic regressions allow for the examination of the associations between the independent variables and each race outcome, Black or Another race, compared to the reference outcome, White. Unlike Ordinary Least Squares (OLS) and logistic regressions, multinomial logistic regressions can handle outcome variables with more than two levels and does not assume normality, linearity, or homoscedasticity. We calculated the VIF for each predictor variable. The results showed that all VIF values were below the threshold of ten, indicating no significant multicollinearity and the independent variables in the analysis were not too closely related to each other.

To address the study’s research question and capture heterogeneity within Latinx populations, we stratified the multinomial logistic regression models by nationality group, including Mexican, Cuban, Puerto Rican, Dominican, and Central/South American adults (see Tables 3, 4). As mentioned in the introduction, Latinx populations are not a monolith and vary in their historical contexts, migration patterns, socioeconomic statuses, racialization experiences. By running stratified models, we can capture the unique influences and associations within each nationality group without assuming homogeneity across all Latinx individuals. Combining all nationality groups into a single model could mask subgroup-specific effects and lead to aggregation bias. Stratified models help prevent such biases by analyzing each subgroup separately. To address reasonable caution that emerges in the form of unobserved heterogeneity when using stratified models, we followed Carina Mood’s (2010) recommendation to account for unobserved heterogeneity. Therefore, we estimated the Average Marginal Effects (AME) for the independent variables of immigrant status and SES after each multinomial regression model of each nationality group (see Tables 5, 6). Due to AMEs being a standardized coefficient, it allows for the comparison of the size of the effect of the predictors between models. Prior to running the multinomial logistic regressions for each nationality group, we ran it for the entire Latinx sample that included each nationality group, the panethnic Latinx model (see Table 2), which provides further support for the differences in effect size for all predictors in one model with the entire sample.

**Table 2.** Adjusted Odds Ratio (aOR) and AME of Racial self-classifications among Panethnic Group

	Black (vs. White)	Black (vs. White)	Another Race (vs. White)	Another Race (vs. White)
	aOR (CI)	AME (SE)	aOR (CI)	AME (SE)
**Independent variables**				
**Immigrant status**				
Recent immigrant	Ref	Ref	Ref	Ref
Long-term immigrant	1.13 (0.82 – 1.55)	.001 (.002)	1.12* (1.01 – 1.26)	.025* (.012)
US-born	1.59** (1.13 – 2.23)	.011 (.003)	.66*** (.58– .76)	−.089*** (.015)
**SES**				
High SES	Ref	Ref	Ref	Ref
Low SES	1.18 (0.91 – 1.53)	−.000* (.002)	1.66*** (1.48 – 1.87)	.106*** (.012)
Middle SES	1.02 (0.79 – 1.31)	−.002 (.002)	1.37*** (1.24 – 1.52)	.066*** (.010)
**Nationality**				
Mexican	Ref	Ref	Ref	Ref
Cuban	4.77*** (3.28– 6.95)	.029*** (.004)	.21*** (0.16 – 0.28)	−.261*** (.016)
Puerto Rican	10.83*** (8.05– 14.58)	.050*** (.005)	.87*(0.76 – 1.00)	−.046*** (.015)
Dominican	34.01*** (22.78– 50.80)	.107*** (.015)	2.09*** (1.72 – 2.53)	.112*** (.022)
Central/South American	5.01*** (3.70– 6.79)	.020*** (.003)	1.09* (0.98 – 1.22)	.013 (.012)

Data Source: NCHS, National Health Interview Surveys, 2010–2018.

Note. Model controlled for age, sex, marital status, U.S. region and NHIS year. They are omitted from display and can be available upon request. CI= Confidence interval; Ref = Reference Group AME = Average marginal effect (i.e., discrete difference in the average adjusted predictions). SE = Delta-method standard error. * p<0.05; ** p<0.01; *** p<0.001.

**Table 3.** Adjusted Odds Ratio (aOR) of Black Racial self-classification by Latinx Nationality Group

	Mexican (n=24,620)	Cuban (n= 2,150)	Puerto Rican (n=4,378)	Dominican (1,462)	CSA (6,681)
	Black (vs. White)	Black (vs. White)	Black (vs. White)	Black (vs. White)	Black (vs. White)
	aOR (CI)	aOR (CI)	aOR (CI)	aOR (CI)	aOR (CI)
**Independent variables**					
**Immigrant status**					
Recent immigrant	Ref	Ref	Ref	Ref	Ref
Long-term immigrant	0.63 (0.23 – 1.71)	0.65 (0.30 – 1.40)	1.83 (0.83 – 4.02)	1.14 (0.58 – 2.22)	1.14 (0.56 – 2.30)
U.S.-born	1.41 (0.52 – 3.81)	.97 (0.35 – 2.67)	2.66** (1.28 – 5.55)	0.82 (0.38 – 1.81)	2.79* (1.16 – 6.68)
**SES**					
High SES	Ref	Ref	Ref	Ref	Ref
Low SES	0.73 (0.38 – 1.39)	5.01* (1.40 – 17.99)	1.97** (1.21 – 3.22)	0.70 (0.33 – 1.47)	1.38 (0.80 – 2.36)
Middle SES	0.80 (0.46 – 1.40)	2.67* (1.00 – 7.16)	1.12 (.68 – 1.85)	0.81 (0.40 – 1.61)	1.04 (0.62 – 1.75)

Data Source: NCHS, National Health Interview Surveys, 2010–2018.

Note. Model controlled for age, sex, marital status, U.S. region and NHIS year. They are omitted from display and can be available upon request. CI= Confidence interval; Ref = Reference Group; CSA= Central/South American. * p<0.05; ** p<0.01; *** p<0.001.

**Table 4.** Average Marginal Effects (AME) of Black Racial Self-Classification by Latinx Nationality Group

	Mexican		Cuban		Puerto Rican		Dominican		CSA	
Independent Variables	dy/dx	SE	dy/dx	SE	dy/dx	SE	dy/dx	SE	dy/dx	SE
**Immigrant status**										
Recent immigrant	Ref	Ref	Ref	Ref	Ref	Ref	Ref	Ref	Ref	Ref
Long-term immigrant	−.002	(.002)	−.015	(.015)	.020	(.013)	.016	(.045)	.002	(.007)
U.S.-born	.003	(.003)	.003	(.003)	.045***	(.012)	−.016	(.048)	.040***	(.017)
**SES**										
High SES	Ref	Ref	Ref	Ref	Ref	Ref	Ref	Ref	Ref	Ref
Low SES	−.003	(.003)	.061	(.039)	.030*m*	(.016)	−.063	(.045)	.004	(.014)
Middle SES	−.002	(.002)	.028	(.017)	.002	(.015)	−.029	(.046)	−.003	(.013)

dy/dx = average marginal effect (i.e., discrete difference in the average adjusted predictions). SE = Delta-method standard error; Ref = Reference Group; CSA= Central/South American. * p<0.05; ** p<0.01; *** p<0.001.

**Table 5.** Adjusted Odds Ratio (aOR) of Another Race Racial Self-classification by Latinx Nationality Group

	Mexican (n=24,620)	Cuban (n=2,223)	Puerto Rican (n=4,277)	Dominican (1,462)	CSA (6,681)
	Black (vs. White)	Black (vs. White)	Black (vs. White)	Black (vs. White)	Black (vs. White)
	aOR (CI)	aOR (CI)	aOR (CI)	aOR (CI)	aOR (CI)
**Independent variables**					
**Immigrant status**					
Recent immigrant	Ref	Ref	Ref	Ref	Ref
Long-term immigrant	1.20* (1.02 – 1.41)	.97 (0.55 – 1.71)	1.15 (0.80 – 1.64)	1.03 (0.64 – 1.68)	1.04 (0.85 – 1.26)
U.S.-born	.63*** (0.52 – 0.76)	.64 (0.30 – 1.34)	.90 (0.63 – 1.28)	0.80 (0.48 – 1.33)	.73* (0.56 – .94)
**SES**					
High SES	Ref	Ref	Ref	Ref	Ref
Low SES	1.36*** (1.17 – 1.59)	4.27*** (2.01 – 9.06)	2.29*** (1.74 – 3.02)	1.65 (0.96 – 2.84)	2.21*** (1.76 – 2.78)
Middle SES	1.28*** (1.13 – 1.45)	2.85*** (1.51 – 5.37)	1.39* (1.07 – 1.81)	1.01 (0.58 – 1.75)	1.49*** (1.20 – 1.84)

Data Source: NCHS, National Health Interview Surveys, 2010–2018.

Note. Model controlled for age, sex, marital status, U.S. region and NHIS year. They are omitted from display and can be available upon request. CI= Confidence interval; Ref = Reference Group; CSA= Central/South American. * p<0.05; ** p<0.01; *** p<0.001.

**Table 6.** Average Marginal Effects (AME) of Another Race Racial Self-Classification by Latinx Nationality Group

	Mexican		Cuban		Puerto Rican		Dominican		CSA	
Independent Variables	dy/dx	(SE)	dy/dx	(SE)	dy/dx	(SE)	dy/dx	(SE)	dy/dx	(SE)
**Immigrant status**										
Recent immigrant	Ref	Ref	Ref	Ref	Ref	Ref	Ref	Ref	Ref	Ref
Long-term immigrant	.041*	(.018)	−.000	(.032)	.020	(.035)	.000	(.044)	.006	(.021)
U.S.-born	−.096***	(.021)	−.044	(.038)	−.031	(.033)	−.033	(.043)	−.076**	(.026)
**SES**										
High SES	Ref	Ref	Ref	Ref	Ref	Ref	Ref	Ref	Ref	Ref
Low SES	.061***	(.015)	.119***	(.036)	.141***	(.025)	.107	(.049)	.154***	(.023)
Middle SES	.048***	(.012)	.080**	(.026)	.054*	(.022)	.010	(.046)	.074***	(.020)

dy/dx = average marginal effect (i.e., discrete difference in the average adjusted predictions). SE = Delta-method standard error; Ref = Reference Group; CSA= Central/South American. * p<0.05; ** p<0.01; *** p<0.001.

## Results

### Characteristics of Total Sample

According to the descriptive analyses as shown in Table 1, the racial self-classifications of Latinx adults in the total sample group closely resembles how Latinx individuals reported their race in the 2010 Census. Most respondents (61%) classified as White, followed by Another race (38%) and Black (2%). Forty-nine percent of respondents have been living in the United States for more than ten years and a slightly higher percentage were born in the United States (41%). Slightly over half of participants are middle SES (51%), one-third are low SES (33%), and fifteen percent are high SES. The majority of respondents (64%) reported having a Mexican ethnic background, followed by Central/South American (18%), Puerto Rican (10%), Cuban (5%), and Dominican (4%). Most respondents were between the ages of thirty-five and forty-nine, lived with a partner or were married (61%), and reported living either in the U.S. West (40%) or South (37%). Finally, there was an even distribution of sex (male vs. female).^6^

### Characteristics by Racial Self-classification

Most WhiteLatinx adults in this study were Mexican (63%), were either born in the United States (45%) or were long-term immigrants (45%), were middle SES (52%), and resided in the U.S. South (44%) or West (33%). Similar to Latinxs of Another race, most WhiteLatinxs were cohabitating/married versus single (62%, 38%). Majority of AfroLatinx adults in the study are Puerto Rican (36%), U.S.-born (51%), middle SES (47%). More AfroLatinxs were women versus men (52%, 48%) and single versus cohabitating/married (55%, 45%). Most AfroLatinxs live in the U.S. South or Northeast (38%, 37%). Unlike their White and Black peers, most Latinxs who racially classified as Another race were born outside the United States, most being long-term immigrants (55%). More Latinxs of Another race occupy the low SES class (39%). Most live in the U.S. West (52%). Like WhiteLatinx adults, most respondents of Another race were Mexican (68%).

### Factors of Racial Self-classification among Panethnic Latinx Adults

Table 2 displays the adjusted odds ratio (aOR) of immigrant status, socioeconomic (SES), and nationality for each racial self-classification outcome compared to White. These models controlled for U.S. region, age, sex, marital status, and survey year.

#### Immigrant Status

Immigrant status was significantly associated with racial self-classification for both Black and Another race. Compared to recently immigrated respondents, U.S.-born respondents had 59% higher odds of reporting Black as their race over White (aOR = 1.59, 95% CI [1.14 – 2.23]). Conversely, U.S.-born respondents had 34% lower odds of reporting Another race over White (aOR = 0.66, 95% CI [0.58 – 0.76]).

#### Socioeconomic Status

Though SES was not significantly associated with Black racial self-classification, it was significantly associated Another race. Compared to high SES respondents, low and middle SES Latinx adults had higher odds (67%, 37%, respectively) of reporting Another race (aOR = 1.67, 95% CI [1.48 – 1.87]; aOR = 1.37, 95% CI [1.25 – 1.52]).

#### Nationality

Nationality was also a significant predictor of racial self-classification. Compared to Mexican respondents, Cuban, Puerto Rican, Dominican, and Central/South American adults had higher odds (377%, 983%, 2401%, 401%, respectively) of reporting Black racial self-classification (aOR= 4.77, 95% CI [3.28 – 6.95]; aOR= 10.83, 95% CI [8.05 – 14.58]; aOR= 34.01, 95% CI [22.78 – 50.80]; aOR= 5.01, 95% CI [3.70 – 6.79]). For Another race, Cuban and Puerto Rican respondents had lower odds (79%, 21%, respectively) compared to Mexican respondents (aOR= 0.21, 95% CI [0.15 – 0.29]; aOR= 0.79, 95% CI [0.67– 0.93]). However, Dominican respondents had 78% higher odds of reporting Another race over White (aOR= 1.78, 95% CI [1.43 – 2.21]). The association between Central/South American nationality and Another race was not significant.

### Factors of Racial Self-classification by Latinx Nationality Group

Tables 3–6 display the adjusted odds ratios (aOR) and average marginal effects of immigrant and SES factors on the probability of reporting Black or Another race compared to White for each Latinx nationality group included in this study (i.e., Mexicans, Cubans, Puerto Ricans, Dominicans, and Central/South Americans).

#### Immigrant Status

Immigrant status was significantly associated with Black racial self-classification among Puerto Rican and Central/South American adults but not among Mexican, Cuban, or Dominican respondents. Puerto Rican adults born in the United States had 166% higher odds of reporting Black as their race over White compared to their recently migrated peers (aOR= 2.66, 95% CI [1.28 – 5.55]). The AME indicates being born in the United States, compared to recently immigrating and having lived less than ten years in the United States, increases the probability of identifying as Black by 4.5% for Puerto Rican adults. Central/South American adults born in the U.S. had 179% higher odds of reporting Black as their race over White compared to recent immigrants (aOR= 2.79, 95% CI [1.16 – 6.68]). The AME shows that being born in the United States increases the probability of identifying as Black by 4 % for Central/South American adults.

Immigrant status was significantly associated with Another race only among Mexican and Central/South American adults. Mexican adults born in the United States had 37% lower odds of reporting their race as Another race over White compared to recent immigrants (aOR= 0.63, 95% CI [0.52 – 0.76]). AME indicates that being U.S.-born decreases the probability of identifying as Another race by 9.6%, on average for Mexican adults. Central/South American adults born in the United States had 27% lower odds of reporting their race as Another race over White (aOR= 0.73, 95% CI [0.56 – 0.94]). AME indicates being born in the United States, compared to recently immigrating and having lived less than ten years in the United States, decreases the probability of identifying as Another race by 7.6%, on average for Central/South American adults.

#### Socioeconomic Status

SES class was significantly associated with racial self-classification among all ethnic groups except Dominican respondents.

Among Puerto Rican adults, low SES class was associated with higher odds of both Black and Another race classifications over White (aOR= 1.97, 95% CI [1.21 – 3.22] for Black; aOR= 2.29, 95% CI [1.74 – 3.02] for Another race). Middle SES class was also significantly associated with higher odds of Another race over White (aOR= 1.39, 95% CI [1.07 – 1.81]). AME indicates being low SES class increases the probability of identifying as Black by 3.0% and Another race by 14.1%. AME also indicates that being in a middle SES class increases the probability of identifying as Another race by 5.4%.

Among Cuban adults, low SES class was significantly associated with higher odds of both Black and Another race classifications over White (aOR= 5.01, 95% CI [1.40 – 17.99] for Black; aOR= 4.27, 95% CI [2.01 – 9.06] for Another race). Middle SES class was also significantly associated with higher odds of Another race over White (aOR= 2.85, 95% CI [1.51 – 5.37]). AME indicates that being in a low SES class increases the probability of identifying as Black by 6.1% and as Another race by 11.9%. AME also indicates that being in a middle SES class increases the probability of identifying as Another race by 8%.

For Mexican adults, both low and middle SES classes were significantly associated with higher odds of Another race racial self-classification compared to high SES respondents (aOR= 1.36, 95% CI [1.17 - 1.58] for low SES; aOR= 1.28 for middle SES, 95% CI [1.13 - 1.45] for middle SES). AME indicates that low and middle SES class increases the probability of identifying as Another race by 6.1% and 4.8%, respectively.

For Central/South American adults, both low and middle SES classes were significantly associated with higher odds of Another race classification over White (aOR= 2.21, 95% CI [1.76 – 2.78] for low SES; aOR= 1.49, 95% CI [1.20 – 1.84] for middle SES). AME indicates that low and middle SES class increases the probability of identifying as Another race by 15.3% and 7.4%, respectively.

## Discussion

This study assessed the associations between immigrant status, socioeconomic status (SES), and racial self-classification among Latinx adults from diverse nationalities, contributing to an underexplored area of research. Findings reveal that these predictors influence Black and Another race self-classification differently across nationality groups, challenging prevailing assumptions and enriching theoretical frameworks around racial identity development.

### Immigrant Status and Black Identity

Immigrant status emerged as a significant predictor of Black racial self-classification among the Latinx adults. Being born in the United States was associated with an increased probability of self-classifying as Black. When examining across nationality groups, it was a significant predictor for Puerto Rican and Central/South American adults but not for other groups. This finding challenges traditional assimilation theories and the social Whitening hypothesis, which suggests longer length of U.S. residence would increase the likelihood of White identification. Instead, these results align with frameworks emphasizing segmented racialization processes. Being born in the United States may expose Puerto Ricans and Central/South Americans to unique racialization experiences, such as being socially ascribed and treated as Black. This could lead to heightened awareness of Blackness, anti-Black racism, and a consequent recognition of not being White. Black racial consciousness has been linked to greater salience of Black racial identity (Sellers 1998). For instance, the Black Lives Matter movement led many AfroLatinxs to explicitly embrace their Black identity and express a shared fate with non-Latinx African Americans, joining in solidarity as members of the Black diaspora (Francis 2021; Hordge-Freeman 2021). These results also align more closely with studies suggesting island-born Puerto Ricans prefer a White racial identity compared to those with fewer ties to Puerto Rico (Vargas-Ramos 2015). The stigma of Blackness in Latin America may produce a strong aversion to self-classifying as Black among recently immigrated Latinx adults (Darity et al., 2005; Haywood 2017).

The observed increased probability in Black self-classification among U.S.-born Puerto Ricans may be related to closer proximity to Blackness, such as membership in a multiracial family and/or a predominantly Black neighborhood. Research has shown that U.S.-born Puerto Ricans are more likely to marry a non-Latinx Black partner (De Jesús et al., 2014). Therefore, those born in the United States may be more likely to have one Puerto Rican parent and one non-Latinx Black parent, which could influence their Black racial identity development. Additionally, Puerto Ricans are more likely to reside in predominantly African American neighborhoods compared to other Latinx groups, influenced by a combination of socioeconomic factors, historical context, and shared racial identities (Bottia 2019). These family and residential patterns may expose U.S.-born Puerto Ricans to positive racial socialization dynamics that, in turn, increase pride and embrace of Black identity. For example, evidence shows that parental cultural socialization practices, such as racial pride messages, discussing cultural traditions, and values are associated with increased ethnic-racial identity exploration and affirmation and stronger ethnic-racial identity among Latinx and Black youth (Hughes et al., 2006; Huguley et al., 2019; Umaña-Taylor and Guimond, 2012).

### SES and Racial Self-Classification

SES was a significant predictor of Another race among Latinx adults, but not Black racial self-classification. The nationality stratified models show that SES influences racial self-classification patterns in diverse ways across nationality groups. Low and middle SES Cuban, Puerto Rican, and Mexican adults, compared to their high SES peers, were more likely to identify themselves as Black or Another race than White. One possible explanation may be that these respondents are experiencing Anti-Black or Anti-Latinx racialization, exclusion, and marginalization associated with low SES, potentially fostering stronger identification with Blackness, Latinidad, and a sense of otherness. For example, in the case of Mexicans in the sample, racialization experiences associated with being labeled “Hispanic/Latino” or Mexican, such as racial profiling in states that have strict anti-immigrant laws and policies may prompt racial identification with a panethnic Latino or Mexican identity to affirm cultural heritage and resist imposed racial hierarchies (Dowling 2014). These findings may also indicate a bidirectional relationship. Those who are racialized as Black or Another race may be more likely to face economic disadvantages and be more exposed to low SES conditions (e.g., higher levels of poverty) compared to their White counterparts. For example, structural anti-Black racism may leave AfroCubans and AfroPuerto Ricans with limited or less prestigious job opportunities. Unlike with immigrant status, this finding aligns with the social Whitening hypothesis. Higher SES individuals may align with Whiteness to safeguard social mobility and economic privilege, particularly in predominantly White, conservative environments.

Previous research found that acculturation plays a role in the racial self-classification patterns of Latinxs in the United States (Rodriguez et al., 2013). These findings suggest indicators of acculturation, such as immigrant status, differentially impact how Latinx nationality groups racially self-classify themselves. Acculturation maybe a key mechanism in studying racial identity development among Latinxs in the United States. However, neither immigrant status nor SES was significantly associated with Black self-classification among Mexican or Dominican adults. This may indicate that factors beyond the scope of this study may be more salient in shaping their relationship with Black identity, such as phenotype, skin color discrimination, or internalized racial attitudes. As mentioned earlier in this paper, researchers have documented how experiencing skin color discrimination or colorism may also draw one closer to Black identity and how internalized colorism may draw them closer to identifying more with Whiteness (Stokes-Brown 2012; Adames et al., 2021). For example, while Dominicans in the sample are more likely to self-identify as Black compared to other ethnic groups in the panethnic model, some may still distance themselves from any Black ancestry they may have and prefer to identity with Whiteness due to internalized anti-Black attitudes rooted in *Mestizaje Racial Ideologies*, *blanqueamiento* and *antihaitianismo* (Adames et al., 2021; Lamb and Dundes, 2017). As Tanya K. Hernández (2022) asserts in her book *Racial Innocence: Unmasking Latino Anti-Black Bias and the Struggle for Equality*, anti-Black attitudes and bias within Latinx communities can be both internalized and resisted, particularly among those exposed to U.S. racial dynamics.

### Limitations

A limitation of this study is the reliance on racial self-classification to operationalize respondents’ race. Although racial self-classification is a standard measure of self-reported race used in official surveys, such as the Census, it is unidimensional and does not capture additional dimensions of race. Latinx individuals experience race in diverse ways, including self-perceived identity, ascribed identity, and skin color, which are not encompassed by racial self-classification alone (Roth 2016). This unidimensional measure overlooks complexities such as racial mismatch experiences, where individuals’ self-identified race differs from how others perceive them (Roth 2010; Vargas and Stainback, 2016). These mismatches may influence racial identity and its associated outcomes in ways this study could not account for.

Emerging concepts offer insights into additional dimensions of race that may enhance understanding of Latinx racial identity. Nancy López and colleagues (2018) recently developed and tested a dimension of race tied to how one is racialized by others according to their skin color and phenotype, called “street race”. Using data from the 2015 National Latino Health Survey, they found discrepancies between street race and self-perceived race, demonstrating that fewer Latinx adults are racialized as White compared to those who self-identify as such. Given this and qualitative findings on how colorblind racial attitudes influence racial self-classification (Dowling 2014), racial self-classification may be indirectly reflecting additional constructs, such as skin color, *Mestizaje Racial Ideologies*, and street race.

Furthermore, the absence of skin color and discrimination measures in this study puts into question whether the relationships between immigrant status, SES, and racial self-classification would remain significant if these factors were accounted for in the statistical analyses. Without these additional measures and multiple dimensions of race, this study’s findings are constrained in their ability to fully contextualize the processes shaping racial identity among Latinx individuals.

## Conclusion

This study uniquely contributes to previous research on racial self-classification patterns of Latinxs in the United States by highlighting intra-group diversity across nationality groups, providing further preliminary evidence of segmented racialization processes that challenge homogenizing narratives about Latinx racial identity. The use of stratified models and average marginal effects in the primary analysis allowed us to uncover unique nationality-specific patterns that would have been obscured in aggregated analyses, offering a better understanding of racial identity formation among Latinx adults. We found that immigrant status and SES significantly influenced racial self-classification, with notable variation across Puerto Rican, Cuban, Mexican, Dominican, and Central/South American adults, suggesting that acculturation and segmented racialization processes may operate differently across these nationality groups. However, future research should replicate this study using additional factors that may be stronger predictors, such as skin color, racial attitudes, discrimination, and street race, to deepen our understanding of these relationships and dynamics.

Gaining a better understanding of these potential predictors and moderators of racial identity can inform interventions that address the psychological and social needs of racially diverse Latinx populations. Black racial identity, while fostering solidarity and pride, may also be associated with heightened race-related stress and mental health challenges, particularly for U.S.-born Puerto Ricans and Central/South Americans in this study. Addressing these inequities requires interventions that consider anti-racist frameworks and factors that may influence racial identity development. For example, anti-racist interventions that combat internalized stigma towards Blackness can promote healthy pathways to Black racial identity development (Banks et al., 2021). These interventions may include educational programs that dismantle colorism and anti-Black stereotypes, community-based initiatives promoting AfroLatinx cultural pride, and mental health support tailored to the unique challenges of Black-identifying Latinxs. As Amalia Dache and colleagues (2019) assert in their conceptualization of a “Black-imiento”, embracing Blackness as a “badge of honor” rather than shame can serve as a powerful form of resistance against anti-Black racism in Latinx communities. This framework underscores the importance of centering Blackness and acknowledging the ongoing influence of anti-Blackness within Latinidad, providing a critical foundation for interventions aimed at fostering positive Black racial identity. This is particularly important in light of the increasing visibility and population growth of AfroLatinx individuals (Galdámez et al., 2023).

Moreover, given the critical role of tracking accurate race data in addressing racial inequities, ensuring accurate representation of segmented racialization processes is essential for policy and research. For example, the recent federal policy change made by The Office of Management and Budget (OMB) to combine race and ethnicity into a single Census question risks erasing AfroLatinx identity (Hernández 2024) and racial identities that may be shaped by unique and segmented racialization processes observed in this study. Therefore, policymakers and researchers should consider prioritizing ways to include multiple dimensions of race like skin color and street race to ensure accurate representation. These approaches can enhance the understanding of the inequities faced by minoritized Latinx communities, such as AfroLatinx populations, and inform policies to address disparities in health, economic, and social outcomes.

## Notes

1 We use “Another race” in place of “Some Other Race” to avoid othering language.

2 We use AfroLatinx to refer to an individual who self-classifies as Latinx as their ethnicity and Black as their race.

3
*Mestizaje Racial Ideologies*, as defined by Hector Adames and colleagues (2021), refer to racial attitudes that promote racial mixing as a national identity while simultaneously erasing Blackness and reinforcing anti-Blackness within Latinx communities.

4
https://archive.cdc.gov/www_cdc_gov/nchs/nhis/1997-2018.htm (accessed March 4, 2025).

5
https://ftp.cdc.gov/pub/Health_Statistics/NCHS/Dataset_Documentation/NHIS/2018/srvydesc.pdf (accessed March 4, 2025).

6 The dataset classified sex using binary categories (“male” and “female”). We acknowledge that sex assigned at birth and gender identity are distinct, and that this binary classification does not include intersex or a full spectrum of gender identities. We call on researchers to engage in more inclusive data collection practices that separately ask about sex assigned at birth and gender identity, incorporating intersex, nonbinary, transgender, and gender-expansive options.
